# A Genome-Wide Association Study of Hypertension and Blood Pressure in African Americans

**DOI:** 10.1371/journal.pgen.1000564

**Published:** 2009-07-17

**Authors:** Adebowale Adeyemo, Norman Gerry, Guanjie Chen, Alan Herbert, Ayo Doumatey, Hanxia Huang, Jie Zhou, Kerrie Lashley, Yuanxiu Chen, Michael Christman, Charles Rotimi

**Affiliations:** 1 Center for Research on Genomics and Global Health, National Human Genome Research Institute, National Institutes of Health, Bethesda, Maryland, United States of America; 2 The Coriell Institute for Biomedical Research, Camden, New Jersey, United States of America; 3 Department of Genetics and Genomics, Boston University, Boston, Massachusetts, United States of America; 4 National Human Genome Center, Howard University, Washington, D.C., United States of America; University of Geneva Medical School, Switzerland

## Abstract

The evidence for the existence of genetic susceptibility variants for the common form of hypertension (“essential hypertension”) remains weak and inconsistent. We sought genetic variants underlying blood pressure (BP) by conducting a genome-wide association study (GWAS) among African Americans, a population group in the United States that is disproportionately affected by hypertension and associated complications, including stroke and kidney diseases. Using a dense panel of over 800,000 SNPs in a discovery sample of 1,017 African Americans from the Washington, D.C., metropolitan region, we identified multiple SNPs reaching genome-wide significance for systolic BP in or near the genes: PMS1, SLC24A4, YWHA7, IPO7, and CACANA1H. Two of these genes, SLC24A4 (a sodium/potassium/calcium exchanger) and CACNA1H (a voltage-dependent calcium channel), are potential candidate genes for BP regulation and the latter is a drug target for a class of calcium channel blockers. No variant reached genome wide significance for association with diastolic BP (top scoring SNP rs1867226, p = 5.8×10^−7^) or with hypertension as a binary trait (top scoring SNP rs9791170, p = 5.1×10^−7^). We replicated some of the significant SNPs in a sample of West Africans. Pathway analysis revealed that genes harboring top-scoring variants cluster in pathways and networks of biologic relevance to hypertension and BP regulation. This is the first GWAS for hypertension and BP in an African American population. The findings suggests that, in addition to or in lieu of relying solely on replicated variants of moderate-to-large effect reaching genome-wide significance, pathway and network approaches may be useful in identifying and prioritizing candidate genes/loci for further experiments.

## Author Summary

Despite intense research, the genetic risk factors for essential hypertension and blood pressure (BP) regulation have not been identified with consistency. We conducted a genome wide association scan using over 800,000 genetic markers in an African American sample of 1,017 adults in the Washington, D.C., area of the United States. We found evidence to suggest that genetic variants in several genes, including PMS1, SLC24A4, YWHA7, IPO7, and CACANA1H, are significantly associated with systolic BP levels. From our previous knowledge of human physiology, two of these genes have potential roles to play in BP regulation. The evidence for genetic variants influencing diastolic BP levels and hypertension status was weaker and inconclusive. To our knowledge, this is the first study that has used a genome-wide association approach to study hypertension and BP in an African American population, a minority group that experiences hypertension more frequently and more severely than other population groups in the United States. The findings will be useful to other researchers seeking to advance our understanding of the genetic factors that influence BP with the hope that these insights will eventually translate to new and better treatment options for hypertension in African Americans and other global populations.

## Introduction

Genome wide association studies (GWAS) on large scale population samples have been remarkably successful in uncovering novel susceptibility loci for a wide range of complex human diseases including type 2 diabetes, coronary artery disease, dyslipidemia, breast cancer, obesity-related traits, prostate cancer and Crohn's disease [1]. These notable success stories represent significant advances in the global effort to understanding the genetic basis of common human diseases. However, this has not been the case for hypertension, a common human disease affecting over one billion people worldwide [2] and a major contributor to cerebrovascular accidents, myocardial infarction, congestive cardiac failure and chronic renal disease [3],[4]. The earliest published GWAS that specifically sought associations for hypertension and/or BP traits (the Wellcome Trust Case Control Consortium (WTCCC) [5] and the Diabetes Genetics Initiative (DGI) [6] studies) did not find any genetic variant significantly associated with hypertension at the genome wide level. While these two studies have some limitations, these negative findings have strengthened the notion that multiple rare independent variants may account for a large fraction of BP variation [7], a situation in which GWAS (designed to work best in “common disease, common variant” scenarios) would be less useful. A further note is that these studies were conducted in European populations and it is unknown if similar studies in populations with non-European ancestry would yield different insights.

In the present study, we conducted a GWAS of BP among African Americans enrolled in the Washington DC metropolitan region of the United States. In comparison with other population groups in the United States, African Americans suffer a disproportionate burden of hypertension and its complications. *A priori*, we considered that: (1) Gene variants associated with BP variation among normotensive individuals may not be exactly the same set identified as those associated with persistently elevated blood pressure (i.e. “hypertension”); (2) Since the clinical definition of hypertension utilizes elevation of either the systolic blood pressure (SBP) or diastolic blood pressure (DBP), those with hypertension are a heterogenous group comprising those with isolated SBP elevation, those with isolated DBP elevation and those with both. This heterogeneity is likely to be reflected in genetic associations for each of these traits (SBP, DBP, hypertension); (3) Individual response to hypertension treatment varies greatly thereby making it a real possibility that statistical adjustment of SBP and DBP for treatment (e.g. adding a fixed quantity to measured BP) among *treated* hypertensive individuals [8], may mask real associations in GWAS. (4) The evidence so far from GWAS of hypertension and BP suggest that there may be few or no variants with large effects, implying that p values may be modest compared to those reported for other traits. For these reasons, we chose to: 1) conduct a case-control association study for hypertension; 2) conduct an association study for SBP and DBP among normotensive individuals; 3) use pathway-based analyses of the GWAS data to determine if the variants most strongly associated with BP phenotypes cluster in pathways and networks that are of biological relevance to BP regulation. Using this strategy, we hoped to maximize the chances of discovering loci influencing hypertension susceptibility and/or normal BP control.

## Methods

### Ethics statement

Ethical approval for the study was obtained from the Howard University Institutional Review Board (IRB). All subjects provided written informed consent for the collection of samples and subsequent analysis. This study was conducted according to the principles expressed in the Declaration of Helsinki.

### Study sample

The subjects studied were all participants in the Howard University Family Study (HUFS), a population based family study of African Americans in the Washington metropolitan area. The major objectives of the HUFS were to: 1) enroll and examine a randomly ascertained cohort of African-American families, along with a set of unrelated individuals, from the Washington DC metropolitan area to study the genetic and environmental basis of common complex diseases including hypertension, obesity and associated phenotypes; 2) to characterize study participants for anthropometry (including weight, height, waist and hip circumferences, body composition measures) and BP; and 3) evaluate the association between genetic variants and selected traits (hypertension, BP and obesity). Participants were sought through door-to-door canvassing, advertisements in local print media and at health fairs and other community gatherings. In order to maximize the utility of this cohort for the study of multiple common traits, families were not ascertained based on any phenotype. During a clinical examination, demographic information was collected by interview. Weight, height, waist circumference and hip circumference were measured using standard methods as follows: Weight was measured in light clothes on an electronic scale to the nearest 0.1 kg, and height was measured with a stadiometer to the nearest 0.1 cm. Body mass index (BMI) was computed as weight in kg divided by the square of the height in meters. Waist circumference was measured to the nearest 0.1 cm at the narrowest part of the torso as seen from the anterior aspect. BP was measured in the sitting position using an oscillometric device (Omron). Three BP readings were taken with a ten minute interval between readings. The reported SBP and DBP readings were the average of the second and third readings. Pulse pressure (PP) was calculated as the difference between the SBP and DBP. Hypertension status was defined as SBP> = 140 mmHg and/or DBP> = 90 mmHg and/or treatment with antihypertensive medication. In the overall cohort, the frequency of hypertension was 35% and among those that were hypertensive, 64% were on antihypertensive medication at the time of the study.

### Genotyping

Genome-wide genotyping was performed using the Affymetrix® Genome-Wide Human SNP Array 6.0 [9]. DNA samples were prepared and hybridized following the manufacturer's instructions. After processing, chips were scanned and genotype calls were made using the Birdseed 2 algorithm [9],[10]. All samples used in the analysis achieved a chip wide call rate of ≥95%. Individual SNPs were excluded if they had a call rate of less than 95% (n = 41,885) across all individuals, a minor allele frequency < = 0.01 (n = 19,154) or had a Hardy-Weinberg equilibrium (HWE) test p of <1×10^−3^ (n = 6,317). The current analysis focused on the 808,465 autosomal SNPs that passed these filters. The average call rate for this set of SNPs in these individuals was 99.5%. The concordance of blind duplicates was 99.74%.

Focused, lower-throughput genotyping for replication was carried out using Sequenom Homogenous MassEXTEND or iPLEX Gold SBE assays at the National Human Genome Research Institute (NHGRI).

### Check for population stratification

Evidence for population stratification or structure was sought by conducting non parametric clustering of genotypes using the AWClust algorithm [11]. All the subjects formed one cluster with a few outliers. Individuals identified as outliers were removed before association analysis, which in this case resulted in the removal of 7 individuals from a sample of 1024 individuals, for a final sample size of 1017 individuals.

Further checks were conducted during the association analysis on the 1017 participants as follows: first, the genomic control (GC) method was used to compute the genomic inflation factor for each analysis and was determined to be 1.007 for hypertension, 1.001 for SBP and 0.998 for DBP, showing minimal evidence of inflation of the test statistic due to stratification. As expected, the GC-adjusted test statistics were virtually identical to the unadjusted values. Second, a Q-Q plot was used to visualize the distribution of the test statistic for each trait analysis and these again showed no evidence of population stratification. Finally, principal components (PC) were computed using the *eigenstrat* method [12]. Based on examination of the scree plot (shown in Figure S1), the first two PCs were retained and used as covariates during the association analysis in order to adjust for any potential residual population stratification.

### Association analyses

Hypertension was analyzed as a binary trait (cases versus controls) using a logistic regression model under an additive model with adjustment for age, sex, BMI, and the first 2 PCs of the genotypes. Given that treatment for hypertension alters BP values, we conducted the association analysis for SBP and DBP in two ways. First, a normotensives-only analysis was carried out using linear regression models with age, sex, BMI, and the first 2 PCs of the genotypes as covariates. This approach was designed to uncover any BP associated loci without the “noise” effect of treatment. Second, an analysis of the whole dataset was carried out using the same covariates and also adjusting for the effect of treatment. All association analyses were performed using the PLINK software package, v1.04 [13]. Association for the replication sample of 980 unrelated non-diabetic West Africans enrolled as part of the Africa America Diabetes (AADM) Study [14],[15] was done the same way. P-values for the discovery (African American) sample and the replication (West African) samples were combined using the Meta-Analysis Tool for genome-wide association scans, *METAL* (http://www.sph.umich.edu/csg/abecasis/Metal/). The METAL algorithm calculates a z-statistic for each marker summarizing the magnitude and direction of the effect relative to the reference allele in each sample and then calculates an overall z-statistic and p value from the weighted average of the statistics. Weights are proportional to the square-root of the sample size of each study.

### Pathway analysis

SNPs that showed an association p-value less than 1e-^04^ for each trait were mapped to genes within 5 kB using Ensembl (http://www.ensembl.org). The resulting gene list for the hypertension phenotype and for SBP and DBP, each with corresponding Entrez IDs, were entered into MetaCore (http://www.genego.com) and tested for enrichment in Maps, Diseases, Gene Ontology (GO) processes and GeneGO processes. MetaCore uses a hypergeometric model to determine the significance of enrichment.

## Results

The subjects comprised 1017 individuals (419 men, 598 women), including 509 cases of hypertension and 508 normotensive controls. Hypertensive subjects were older (mean age 54 years versus 41 years) and heavier (mean BMI 31.7 kg/m^2^ versus 29.3 kg/m^2^) than the normotensive subjects. As expected, mean BP was higher and showed more variance among hypertensive compared to normotensive subjects (Table 1).

**Table 1:** Characteristics of the subjects.

Characteristic	Hypertension	Normotensive controls
N	509	508
Men∶Women	209∶300	210∶298
Age (years)	54.1 (11.8)	42.6 (11.9)
Body mass index (BMI)	31.7 (8.6)	29.3 (8.0)
Waist-hip ratio	0.88 (0.09)	0.85 (0.08)
Systolic BP	144.6 (22.1)	118.0 (10.9)
Diastolic BP	88.2 (13.9)	74.5 (8.0)

Figures are mean (SD).

**Figure 1:**
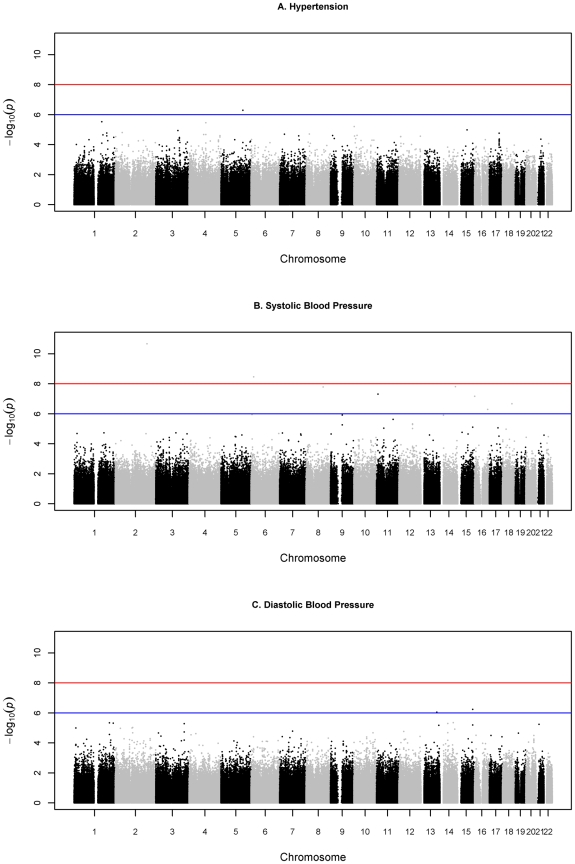
Manhattan plot of all SNPs for the three phenotypes.

**Figure 2:**
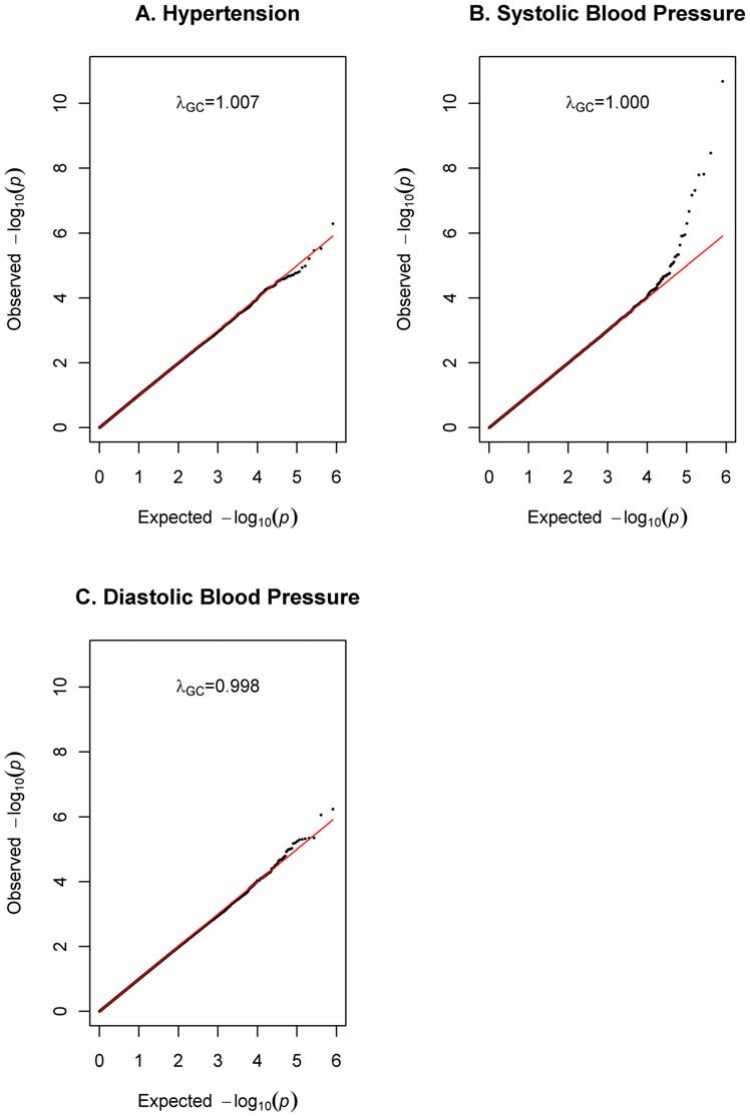
QQ plot for all three phenotypes. QQ plot for hypertension (blue), systolic BP (purple) and diastolic BP (red). Genomic control λ was 1.007 for hypertension, 1.000 for systolic BP and 0.998 for diastolic BP.

The distribution of association p-values (Manhattan plot) for the three traits is shown in Figure 1 and the QQ plots in Figure 2. The ten top scoring SNPs for association with hypertension are shown in Table 2. The SNP with the lowest p-value (5.10×10^−7^) for this trait was rs9791170 located on chromosome 5. This intergenic SNP is about 6 kbp upstream of the P4HA2 (GeneID 8974) gene. However, it did not show genome-wide significance (Bonferroni-corrected p = 0.412) for association with hypertension; neither did any of the other SNPs (see Table S1 for a list of the top-scoring associations for hypertension as a binary trait).

**Table 2:** Top associated SNPs for hypertension as a binary trait.

#	SNP	Chr	Position (bp)	Type	Closest gene	Distance to gene (kb)	A1	MAF	P	OR (SE)
1	rs9791170	5	131597526	INTERGENIC	P4HA2	6	A	0.434	5.10e-07	0.58(0.11)
2	rs2146204	1	167140723	INTERGENIC	RP11-375F2.1	6	C	0.087	2.97e-06	2.49(0.20)
3	rs991316	4	100541468	INTERGENIC	ADH7	11	T	0.451	3.45e-06	1.62(0.10)
4	rs7902529	10	2288418	INTERGENIC	AL354747.12	57	A	0.141	6.14e-06	0.50(0.15)
5	rs1550576	15	56000706	INTERGENIC	ALDH1A2	32	T	0.142	1.03e-05	0.52(0.15)
6	rs11714139	3	133427327	INTERGENIC	ACPP	92	T	0.081	1.14e-05	2.45(0.20)
7	rs11692045	2	41804457	INTERGENIC	LDHAL3	96	C	0.408	1.54e-05	0.64(0.10)
8	rs12748299	1	197249947	INTERGENIC	AC096631.2	64	C	0.132	1.66e-05	1.99(0.16)
9	rs2665797	17	59276217	UPSTREAM	SMARCD2	3	G	0.095	1.73e-05	0.45(0.18)
10	rs11988036	8	20184487	INTRONIC	LZTS1	0	T	0.243	1.95e-05	1.66(0.12)

A1 = minor allele; MAF = minor allele frequency; OR = Odds ratio for minor allele.

In contrast to the hypertension results, the T allele of the rs5743185 SNP, an intronic SNP in the PMS1 (GeneID 5378) gene, was strongly associated with SBP (nominal p = 2.09×10^−11^, Bonferroni-corrected p = 1.69×10^−5^) among normotensive individuals. Other SNPs that showed significant association with SBP among normotensive individuals, each with a Bonferroni-corrected p value of ≤0.05, include: rs3751664 (a non-synonymous coding SNP in CACNA1H (GeneID 8912)), rs11160059 (an intronic SNP inSLC24A4 (GeneID 123041)), rs17365948 (an intronic SNP in YWHAZ (GeneID 7534)), rs12279202 (an intronic SNP in IPO7 (GeneID 10527)) and rs1687730 (an intergenic SNP, 12 kb from AL365365.23, a pseudogene), – Table 3. Repeating these analyses for the whole sample, with adjustment for treatment effects, did not change the top-scoring characteristics of these six SNPs (as shown in Table S2). The mean effect size on SBP associated with the at-risk alleles of these six SNPs (estimated from the linear model adjusted for age, sex, BMI and PCs among normotensive individuals only) was ∼5–6 mmHg. If independent, each SNP significant after Bonferroni-correction correction would be associated with ∼5% of the variance in SBP. The full list of the top-scoring associations for SBP is shown in Table S3. Haplotype analysis did not show any haplotype association that reached the significance of the single locus analyses (data not shown). Two-locus interaction analyses between the SNPs that were significant or marginally so did not show any significant interactions, with the lowest p-value 0.115 (between rs17315498 and rs11160059). For DBP, the A allele of rs1867226 (an intronic SNP in PRC1 (GeneID 9055)) showed the lowest p-value (5.8×10^−7^). However, neither this nor any other association reached genome wide significance (Table 3; see Table S4 for list of top-scoring SNPs for DBP).

**Table 3:** Top associated SNPs for Systolic BP and Diastolic BP.

Rank	SNP	Chr	Position	Type	Closest gene	Distance to gene (kb)	Allele	MAF	P
**Systolic BP**									
1	rs5743185	2	190446083	INTRONIC	PMS1	0	T	0.1418	2.09E-11
2	rs16877320	6	16031005	INTERGENIC	AL365265.23	12	G	0.1316	3.42E-09
3	rs11160059	14	91877083	INTRONIC	SLC24A4	0	A	0.1782	1.54E-08
4	rs17365948	8	102026053	INTRONIC	YWHAZ	0	A	0.1125	1.59E-08
5	rs12279202	11	9388666	INTRONIC	IPO7	0	A	0.1231	4.80E-08
6	rs3751664	16	1194370	NON_SYNONYMOUS_CODING	CACNA1H	0	T	0.1093	6.71E-08
7	rs11659639	18	56318592	INTERGENIC	MC4R	127	C	0.09771	2.13E-07
8	rs4613079	16	79201458	INTRONIC	CDYL2	0	T	0.1766	5.06E-07
9	rs13201744	6	6071844	INTERGENIC	F13A1	17	A	0.16	1.12E-06
10	rs2183737	9	70431453	INTERGENIC	RP11-274B18.3	15	T	0.4592	1.21E-06
**Diastolic BP**									
1	rs1867226	15	89324717	INTRONIC	PRC1	0	C	0.4636	5.80E-07
2	rs9590141	13	94401623	INTERGENIC	ABCC4	68	A	0.1224	8.76E-07
3	rs10135446	14	79479231	INTERGENIC	NRXN3.	79	A	0.1298	4.47E-06
4	rs11120313	1	212647829	INTRONIC	PTPN14	0	A	0.1608	4.53E-06
5	rs16848861	1	235211537	INTERGENIC	RP11-182B22.4	0	G	0.2008	4.73E-06
6	rs11846013	14	46002041	INTERGENIC	RPL10L	188	A	0.136	4.99E-06
7	rs16853574	3	170562457	INTERGENIC	MDS1	19	C	0.03982	5.10E-06
8	rs2823756	21	16664201	INTRONIC	AP000473.2	0	T	0.4389	5.73E-06
9	rs8039294	15	89544863	INTRONIC	SV2B	0	G	0.4828	6.29E-06
10	rs9301196	13	106645433	INTRONIC	FAM155A	0	T	0.1159	6.66E-06

**Figure 3:**
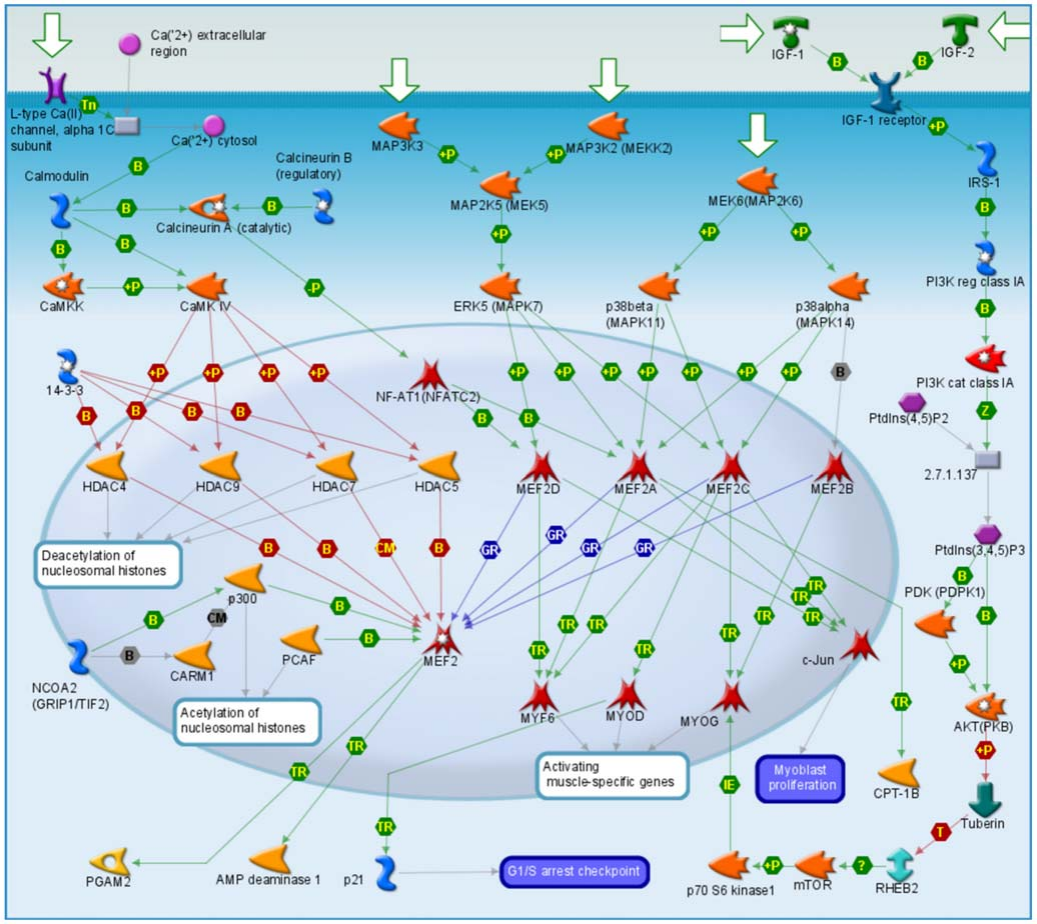
Most significant GeneGo Pathway Map for top scoring genes for SBP and DBP.

Pathway analysis revealed a number of significant pathways and processes that are associated with SBP and DBP (Table 4). Examination of each of these pathways and processes showed annotations with obvious cardiovascular implications (for example, *Development_PIP3 signaling in cardiac myocytes, Transport_Potassium transport and Development_Blood vessel morphogenesis*) and several pathways and processes that are enriched for genes involved in hypertension and/or blood pressure regulation. As a case in point, the top scoring pathway – *Development_Role of HDAC and calcium/clamodulin-dependent kinase (CaMK) in control of skeletal myogenesis*- (Figure 3) contains the calcium-gated channels CACNA1E and CACNA1H, IGF-1, and AKT, each of which is known to play a role in mechanisms of BP regulation, hypertension and/or complications of hypertension (including left ventricular hypertrophy) [16]–[21]. The top-scoring pathways for hypertension alone are shown in Table S5.

**Table 4:** Statistically significant GeneGo Pathway Maps and Biological Processes for genes associated with SBP and DBP at P<1e-05.

GeneGo Pathway Map	p
Development_Role of HDAC and calcium/calmodulin-dependent kinase (CaMK) in control of skeletal myogenesis	2.292e-5
Development_PIP3 signaling in cardiac myocytes	1.179e-3
Development_IGF-RI signaling	1.407e-3
Transcription_Receptor-mediated HIF regulation	3.387e-2
Signal transduction_AKT signaling	4.807e-2
Development_Ligand-independent activation of ESR1 and ESR2	5.277e-2
Signal transduction_PTEN pathway	5.394e-2

GeneGo Biological Process	p
Transport_Synaptic vesicle exocytosis	4.525e-3
Neurophysiological process_Transmission of nerve impulse	1.050e-2
DNA damage_Checkpoint	3.169e-2
Transport_Potassium transport	4.885e-2
Reproduction_FSH-beta signaling pathway	4.986e-2
Cell adhesion_Cell junctions	5.213e-2
Development_Blood vessel morphogenesis	6.740e-2
Signal transduction_Androgen receptor signaling cross-talk	1.250e-1
Signal transduction_ESR1-membrane pathway	1.595e-1
Muscle contraction	2.599e-1

A total number of 17 SNPs were carried forward for replication in the sample of 980 unrelated non-diabetic West Africans (366 HTN cases, 614 normotensive subjects; mean age 49 (SD 12) years, mean BMI 25.1 (SD 6) kg/m^2^) enrolled as part of the Africa America Diabetes Mellitus (AADM) study [14],[15]. These 17 SNPs comprised the top-scoring seven SNPs for SBP, the top scoring three SNPs for DBP, two SNPs that had low p-values (p<1×10^−4^) for both SBP and DBP, as well five of the top-scoring SNPs for HTN as a dichotomous trait. Five (rs5743185, rs3751664, rs12279202, rs11659639 and rs6543012) were monomorphic in the West African sample. The results for the other twelve SNPs analyzed under an additive model and with adjustment for age, sex, BMI, ethnic group and treatment for hypertension (adjustment for treatment for SBP and DBP only) are shown in Table 5. Three SNPs (rs1867226, rs1550576 and rs8039294) were significant at a p-value of <0.05 among the West Africans. The combined analysis showed that five SNPs, including rs11160059 (SLC24A4), were significantly associated with the trait and with the same direction of effect in both samples.

**Table 5:** Replication of selected SNPs in a sample of 980 West Africans.

Chr	SNP (Gene)	Trait	P in African American discovery sample	P in West African replication sample	Combined p value	MAF in African American sample	MAF in West African sample
5	rs9791170	HTN	5.10e-07	0.123	0.009	0.434	0.457
4	rs991316	HTN	3.45e-06	0.081	4.73e-06***	0.451	0.435
1	rs12757682	HTN	2.59e-05	0.410	0.014	0.132	0.079
1	rs12748299	HTN	1.66e-05	0.715	0.0007***	0.132	0.080
15	rs1550576	HTN	1.03e-05	0.039	3.32e-06***	0.142	0.218
6	rs16877320 **(PMS1)**	SBP	3.42e-09	0.781	0.001	0.132	0.023
14	rs11160059 **(SLC24A4)**	SBP	1.54e-08	0.748	0.0003***	0.178	0.078
8	rs17365948 **(YWHAZ)**	SBP	1.59e-08	0.507	0.005	0.113	0.005
15	rs8039294 **(SV2B)**	SBP	7.73e-06	0.038	0.312	0.483	0.354
		DBP	6.29e-06	0.092	0.178		
14	rs10135446	DBP	4.47e-06	0.622	0.002***	0.130	0.141
13	rs9590141	DBP	8.76e-07	0.368	0.031	0.122	0.129
15	rs1867226 **(PRC1)**	DBP	5.8e-07	0.047	0.162	0.464	0.376

***: Same direction of association.

Two recent GWA studies [22],[23] identified the STK39 and CDH13 genes as being significantly associated with BP. We therefore looked for evidence for association of SNPs in these genes with SBP and DBP in the present study. Each of these genes showed multiple SNPs associated with SBP and DBP at a p<0.05 (Table 6). Of note, STK39 had many more significantly associated SNPs (9/136 for SBP, 33/136 for DBP) than would be expected by chance at a nominal p value of 0.05 (7/136). All of the STK39 SBP-associated SNPs and 24 of the 33 DBP-associated SNPs were in the LD bins 1 and 2 (chr2:168,699,002-168,788,544) reported in the Amish.

**Table 6:** Replication of STK39 and CDH13 SNPs with SBP and DBP in this study of African American subjects.

Gene	# SNPs typed	SBP			DBP		
		# SNPs p<0.05	Top SNPs	*P*	# SNPs p<0.05	Top SNPs	*P*
STK39	136	9	rs2063958	0.010	33	rs11890527	1.02×10^−4^
			rs2390639	0.012		rs2203703	2.17×10^−4^
CDH13	1020	49	rs11860907	5.71×10^−4^	51	rs16960421	1.82×10^−3^
			rs7200009	1.08×10^−3^		rs17177428	3.42×10^−3^

We also looked for *in silico* replication of this study's top SBP-associated SNPs in the Diabetes Genetics Initiative (DGI) [6] GWAS, which to our knowledge, was the first published GWAS for BP. Out of the five genes harboring the top scoring SNPs for SBP in this study, three had variants with low p-values associated with SBP under the same additive model in the DGI (Table S6). These were SLC24A4 (rs7142084, p = 0.0017), IPO7 (rs7480643, p = 0.009) and PMS1 (rs3791767, p = 0.014).

## Discussion

Unlike the growing success stories for many common complex diseases (e.g., diabetes), our understanding of the genetic basis of the common type of hypertension (essential hypertension) has not been greatly advanced from the widespread use of GWAS. This is probably due to hypertension being modulated by a larger number of low-risk variants, each of small effect and low penetrance than other complex diseases such as type 2 diabetes. If this is indeed the case, GWAS analysis techniques focusing on identifying common variants of moderate to large effects will not detect these variants, as has been the case for the WTCCC and DGI studies. Another indication that the usual GWAS method may not be as successful for hypertension is the recent attempt to replicate the WTCCC hypertension signals in 11,433 persons in the Family Blood Pressure Program, which essentially could not replicate the WTCC findings [24].

The observation of essentially null findings for hypertension from several large-scale GWAS calls for the development of different approaches including screening for rare variants in genes causing rare diseases characterized by BP change. In the most convincing study using this approach, rare variants in three genes – SLC12A3 (NCCT), SLC12A1 (NKCC2) and KCNJ1 (ROMK) – that alter renal salt handing were shown to influence BP variation in the general population [7]. In the present study, we have used a GWAS as a discovery tool for identifying variants influencing hypertension susceptibility and BP variation among African Americans. Using the set of variants prioritized from the GWAS, our secondary focus was to employ pathway analysis to identify a set of potential genes influencing BP through networks and pathways, rather than to identify a locus or loci of large effect reaching genome-wide significance for association.

Using Bonferroni-corrected p values as the criterion for genome-wide significance, we found significant associations with SBP for several SNPs, comprising one intergenic SNP, four intronic SNPs (in PMS1, SLC24A4, YWHAZ and IPO7) and one non-synonymous coding SNP (in CACANA1H). Two of these genes have annotations that suggest a role in BP regulation. The gene CACNA1H (calcium channel, voltage-dependent, T type, alpha 1H subunit), encodes a T-type member of the alpha-1 subunit family, a protein in the voltage-dependent calcium channel complex. Calcium channels mediate the influx of calcium ions into the cell upon membrane polarization and consist of a complex of alpha-1, alpha-2/delta, beta, and gamma subunits in a 1∶1∶1∶1 ratio. The alpha-1 subunit has 24 transmembrane segments and forms the pore through which ions pass into the cell. The primary disease association is with childhood epilepsy [25]. However, one of its GO annotations is regulation of heart contraction, which provides a plausible mechanism whereby it could influence BP. In fact, it was first cloned from human heart and was shown to be highly expressed in heart and kidney [26]. This T-type voltage-gated calcium channel protein is the target for mibefradil [26], the calcium-channel blocker, used for treating hypertension and angina pectoris under the name *Posicor*. It is also a target for the calcium-channel blocking agents efonidipine, benidipine and manidipine [17]. The associated SNP (rs3751664) is monomorphic in the HapMap Yoruba (YRI) sample but has a minor allele frequency of 0.1 in the HapMap CEU sample which is very similar to the 0.11 frequency observed in this African American sample. This implies that it has its origin in the European component of the African American ancestries. Interestingly, the SNP is a non synonymous coding variant which leads to an arginine to cysteine change in position 788 (R788C) of the protein. This mutation is predicted to be “benign” by *PolyPhen* [27], which may account for its relatively high frequency in the HapMap CEU (0.1), HCB (0.1) and JPT (0.125) samples.

SLC24A4 (solute carrier family 24 (sodium/potassium/calcium exchanger), member 4) codes for potassium dependent sodium/calcium exchanger. Potassium-dependent sodium/calcium exchangers are thought to transport 1 intracellular calcium and 1 potassium ion in exchange for 4 extracellular sodium ions [28]. The associated SNP in the present study, rs11160059, is polymorphic only in the HapMap YRI and is monomorphic in the HapMap CEU, CHB and JPT populations. Interestingly, the only known genetic association with this gene is with hair color and skin pigmentation among people of European ancestry [29],[30]. However, the hair and skin color associated SNP, rs12896399, is located 33 kb away from rs11160059 (the SBP-associated SNP) and both SNPs are not in LD (r^2^ = 0.001 in HapMap ASW and r^2^ = 0.0005 in HapMap YRI). Given the limited functional data available on potassium dependent sodium/calcium exchangers, this gene is worth investigating further as a potential candidate gene for hypertension.

The gene PMS1 (postmeiotic segregation increased 1), encodes a DNA mismatch repair mutL/hexB protein and mutations in this gene cause hereditary nonpolyposis colorectal cancer type 3 (HNPCC3) either alone or in combination with mutations in other genes involved in the HNPCC phenotype (Lynch syndrome). The gene YWHAZ (tyrosine 3-monooxygenase/tryptophan 5-monooxygenase activation protein, zeta polypeptide) codes for one of the 14-3-3 family of proteins which mediate signal transduction by binding to phosphoserine-containing proteins. The encoded protein interacts with IRS1 protein, suggesting a role in regulating insulin sensitivity. IPO7 (importin 7) codes for a protein that is a member of a class of approximately 20 potential Ran targets that share a sequence motif related to the Ran-binding site of importin-beta and is one of several importin beta-like transport receptors, that directly bind and import ribosomal proteins. However, none of these genes is known to have any obvious or direct relationship with BP or hypertension.

As a follow up to the initial GWAS discovery, we carried forward a number of SNPs for replication in a West African sample. Three SNPs showed p of <0.05 and two others a p between 0.05 and 0.1 in the West African sample. However, combined analysis showed that five SNPs - rs991316, rs12748299, rs1550576, rs11160059 (SLC24A4) and rs10135446 – showed a low combined p-value with the same direction of association in both samples. Our search of previously published GWAS for BP for SNPs in the genes represented in the top-scoring SNPs for SBP in the present study also showed that at least one SNP in each of PMS1, SLC24A4 and IPO7 showed low p-values in the DGI study. These findings suggest that these regions are worth further study.

Two recent studies provided the first strong evidence in the literature of genetic variants associated with BP from GWAS. In one, STK39 variants were associated with BP in the Amish population [22] and in the other, CDH13 variants were associated with BP in two European populations [23]. We sought for evidence that variants in these two genes may be associated with SBP in our study of African Americans. Our findings (especially regarding STK39) provide strong supporting evidence that these two genes may indeed be good candidate genes for BP regulation. For each of the genes, the association findings are supported by strong biological evidence: the protein product of STK39 (SPAK) interacts with cation-chloride transporters that play major roles in renal salt excretion and the CDH13 gene codes for an adhesion glycoprotein T-cadherin that is a regulator of vascular wall remodeling and angiogenesis.

We also used pathway-based analysis to provide a means of interpreting the subset of top-scoring SNPs located in genes. Our findings demonstrate that this set of genes cluster in pathways and networks that are likely to be of biological relevance to hypertension and/or BP. A recent study [31] that used this approach on the Wellcome Trust data observed some interesting findings including pathways involved in dopamine signaling, PKA signaling and ChREBP regulation. Indeed, despite the differences between this study and the Wellcome Trust in terms of population studied, study design, phenotypes and sample size, several of the significant pathways were common to both studies (Table S5). Thus, despite the absence of replication at the SNP association level, some gene-based pathway maps were shared between the two studies. This suggests that, in addition to or in lieu of relying solely on replicated variants of moderate-to-large effect reaching genome-wide significance, pathway and network approaches may be useful in prioritizing candidate genes/loci for further experiments [31].

This study has a number of limitations. Firstly, the sample size is modest relative to many other GWAS studies. This means that some signals may have been missed. Secondly, due to lack of GWAS data on African American samples, our replication attempts were limited to a West African sample and *in silico* replication in the DGI study, neither of which may be an appropriate replication sample for African Americans. On the other hand, this is one of the first GWAS for hypertension and BP in a population of non-European ancestry and it increases our resources for understanding the genetics of human hypertension. In this regard, it is important to consider the issue of an appropriate replication sample for an admixed population, such as African Americans. In the absence of any previous published GWAS of hypertension and/or BP in an African American population, we followed the top-scoring SNPs by genotyping a West African sample and showed that two SNPs are worth further study. We also attempted *in silico* replication in a European ancestry sample for our top SBP SNPs (assuming that these two groups represented a significant proportion of the ancestral gene pool of the admixed group). Furthermore, data from the HapMap showed that four of the six SNPs were monomorphic in at least one of these two source populations (represented by the HapMap CEU and YRI samples), suggesting that neither one of these samples will on its own serve as an appropriate replication sample for an African American population. Therefore, the possibility remains that using similar African American populations (who will have similar allele frequencies and haplotype structures) for replication may be optimal, as has recently been reported for East Asian populations and type 2 diabetes [32]. Admixture affects allele frequencies at many loci as well as local LD patterns; furthermore, admixed populations are often not homogenous and may show considerable geographic variation in the degree of admixture. These observations have been found to be true for African Americans, our study population [33],[34]. Therefore, the question of replicating the findings from an admixed population needs to be investigated further.

In summary, we have reported a GWAS for hypertension and BP in an African American population and identified SNPs reaching genome wide significance for SBP and suggestive evidence of association for DBP and Hypertension. The set of top scoring SNPs were enriched for genes in pathways with annotations to hypertension and/or BP regulation. These findings provide a set of candidate genes to be evaluated in-depth in future studies. Further replication and fine mapping in multiple populations, especially in an independent African American samples, are needed. Given the global lack of success in identifying susceptibility loci for essential hypertension using genome wide linkage and association strategies, the genetic architecture underlying BP control must be considerably more complex and sufficiently different from those of other common complex human diseases (e.g., diabetes). This may be the reason why the current agnostic approaches of searching the genome of thousands of individuals for risk loci has not yielded strong and consistent results for hypertension and BP. Alternative strategies, e.g., complete re-sequencing of candidate genes to identify rare variants, are clearly needed.

### Note added during the preparation of the article

While this paper was under review, two GWAS for hypertension, SBP and DBP in subjects of European descent were published [35],[36]. One of these studies [36] also reported finding significant hits in the CACNB2 gene for hypertension and DBP, a gene with a high-scoring variant for hypertension in the present study (Table S1) and in the PMS1 gene for hypertension and SBP, which scored highly for SBP in this study (Table 3).

## Supporting Information

Figure S1 Scree plot of principal components (PCs) of the genotypes in the sample.
(0.13 MB TIF)

Table S1 Top scoring SNPs for association with hypertension as a dichotomous trait.
(0.05 MB XLS)

Table S2 Top scoring SNPs for SBP among all subjects compared to among normotensive subjects (controls) only.
(0.03 MB XLS)

Table S3 Top scoring SNPs for SBP.
(0.05 MB XLS)

Table S4 Top scoring SNPs for DBP.
(0.05 MB XLS)

Table S5 Top scoring GeneGo pathways for top hits for hypertension only.
(0.05 MB DOC)

Table S6 HapMap frequencies and DGI Study SBP p-values in genes for top scoring SBP SNPs. HapMap CEU, YRI, and ASW frequencies for top scoring SBP SNPs. Also shown are the DGI study's lowest p-values in the genes containing the top SNPs for SBP in this study.
(0.03 MB DOC)
