# Supplementary figures and images for: A Genome-Wide Association Study of Hypertension and Blood Pressure in African Americans
Source: PLoS Genet. 2009 Jul 17;5(7):e1000564. doi: 10.1371/journal.pgen.1000564 (PMC2702100; doi:10.1371/journal.pgen.1000564)

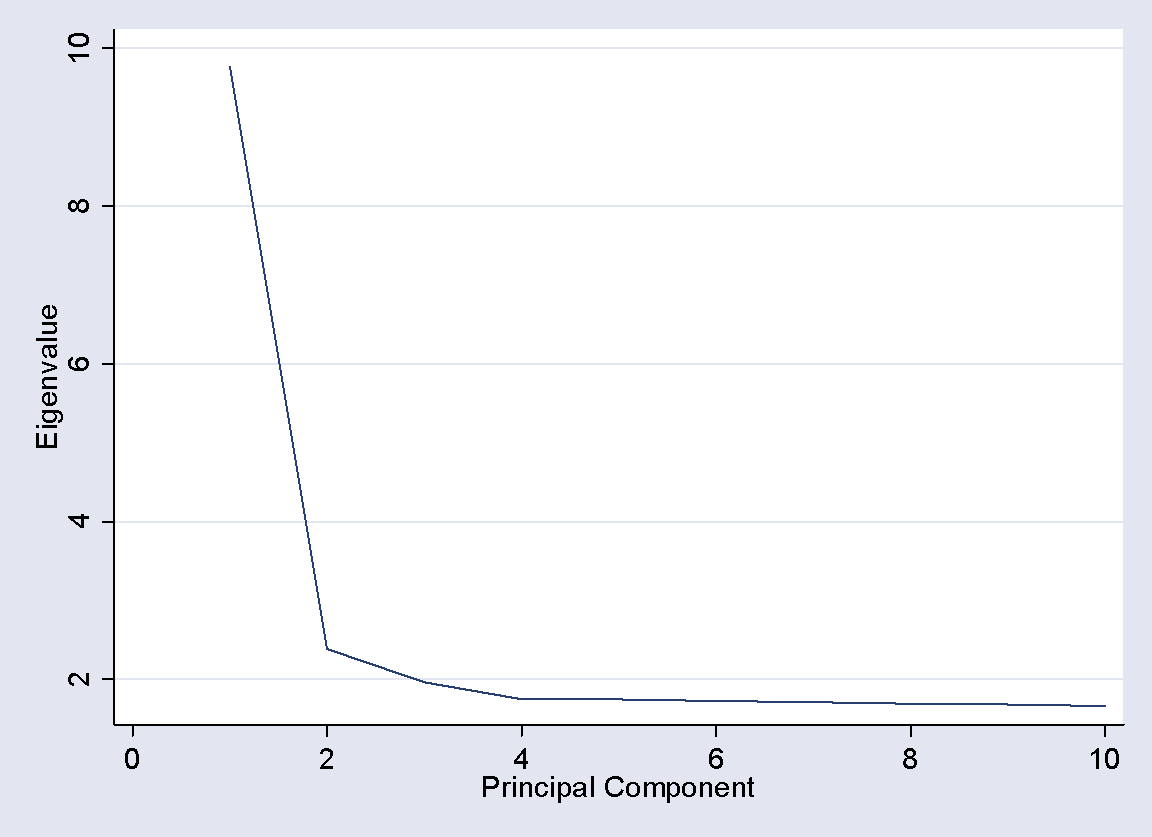

Supplement: Figure S1 — Scree plot of principal components (PCs) of the genotypes in the sample. (0.13 MB TIF) [file pgen.1000564.s001.tif]
