# Supplementary material for: A Genome-Wide Association Study of Hypertension and Blood Pressure in African Americans
Source: PLoS Genet. 2009 Jul 17;5(7):e1000564. doi: 10.1371/journal.pgen.1000564 (PMC2702100; doi:10.1371/journal.pgen.1000564)
Supplement: Table S5 — Top scoring GeneGo pathways for top hits for hypertension only. (0.05 MB DOC) [file pgen.1000564.s006.doc]

GeneGo Pathway maps for top scoring genes for hypertension

| # | Name | pValue |
| --- | --- | --- |
| 1 | G-protein signaling_RAC1 in cellular process | 1.927e-3 |
| 2 | Cell adhesion_Cell-matrix glycoconjugates | 2.704e-3 |
| 3 | [Signal transduction_Erk Interactions: Inhibition of Erk](javascript:enrichments.maps.show_info(447);) | 3.229e-3 |
| 4 | Development_GDNF family signaling | 5.529e-3 |
| 5 | Immune response _CD28 signaling | 6.008e-3 |
| 6 | Development_Prolactin receptor signaling | 8.677e-3 |
| 7 | Transport_Membrane trafficking and signal transduction of G-alpha (i) heterotrimeric G-protein | 4.333e-2 |
| 8 | Immune response _IL22 signaling pathway | 4.333e-2 |
| 9 | [G-protein signaling_Ras family GTPases in kinase cascades (scheme)](javascript:enrichments.maps.show_info(379);) | 5.272e-2 |
| 10 | [Development_EGFR signaling via PIP3](javascript:enrichments.maps.show_info(692);) | 5.272e-2 |
| 11 | [Transcription_ChREBP regulation pathway](javascript:enrichments.maps.show_info(464);) | 5.272e-2 |
| 12 | [Cytoskeleton remodeling_CDC42 in cellular processes](javascript:enrichments.maps.show_info(390);) | 5.505e-2 |
| 13 | [Development_Angiotensin signaling via beta-Arrestin](javascript:enrichments.maps.show_info(547);) | 5.970e-2 |
| 14 | [Neurophysiological process_Dopamine D2 receptor transactivation of PDGFR in CNS](javascript:enrichments.maps.show_info(2455);) | 6.202e-2 |
| 15 | [G-protein signaling_G-Protein alpha-i signaling cascades](javascript:enrichments.maps.show_info(638);) | 6.202e-2 |
| 16 | [Regulation of lipid metabolism_RXR-dependent regulation of lipid metabolism via PPAR, RAR and VDR](javascript:enrichments.maps.show_info(413);) | 6.664e-2 |
| 17 | [Apoptosis and survival_p53-dependent apoptosis](javascript:enrichments.maps.show_info(428);) | 6.894e-2 |
| 18 | [Signal transduction_PKA signaling](javascript:enrichments.maps.show_info(675);) | 6.894e-2 |
| 19 | [Immune response_Delta-type opioid receptor signaling in T-cells](javascript:enrichments.maps.show_info(2665);) | 6.894e-2 |
| 20 | [Transcription_P53 signaling pathway](javascript:enrichments.maps.show_info(412);) | 7.581e-2 |
| 21 | [Development_EGFR signaling via small GTPases](javascript:enrichments.maps.show_info(704);) | 7.581e-2 |
| 22 | [Transcription_Role of AP-1 in regulation of cellular metabolism](javascript:enrichments.maps.show_info(434);) | 7.809e-2 |
| 23 | [Immune response _IL1 signaling pathway](javascript:enrichments.maps.show_info(658);) | 7.809e-2 |
