# Supplementary material for: A Genome-Wide Association Study of Hypertension and Blood Pressure in African Americans
Source: PLoS Genet. 2009 Jul 17;5(7):e1000564. doi: 10.1371/journal.pgen.1000564 (PMC2702100; doi:10.1371/journal.pgen.1000564)
Supplement: Table S6 — HapMap frequencies and DGI Study SBP p-values in genes for top scoring SBP SNPs. HapMap CEU, YRI, and ASW frequencies for top scoring SBP SNPs. Also shown are the DGI study's lowest p-values in the genes containing the top SNPs for SBP in this study. (0.03 MB DOC) [file pgen.1000564.s007.doc]

**Top scoring SNPs for SBP: HapMap frequencies and p-values for SBP in DGI study**

| **Rank** | **SNP** | **Gene** | **HapMap CEU MAF** | **HapMap YRI MAF** | **HapMap ASW MAF** | **DGI GWAS SBP** | | |
| --- | --- | --- | --- | --- | --- | --- | --- | --- |
|  |  |  |  |  |  | **Smallest P in gene** | **SNP** | **Position** |
| 1 | rs5743185 | PMS1 | 0.092 | **0** | - | **0.014** | rs3791767 | 190348160 |
| 2 | rs16877320 | ---------- | 0.042 | 0.033 | 0.012 | ----- | ------ | ------ |
| 3 | rs11160059 | SLC24A4 | **0** | 0.117 | 0.083 | **0.0017** | rs7142084 | 91892784 |
| 4 | rs17365948 | YWHAZ | 0.017 | **0** | - | 0.358 | rs17365305 | 102002858 |
| 5 | rs12279202 | IPO7 | 0.083 | 0.020 | - | **0.009** | rs7480643 | 9388462 |
| 6 | rs3751664 | CACNA1H | 0.1 | **0** | 0.012 | 0.256 | rs909921 | 1213804 |

Minor allele frequency (MAF) of 0 means the locus is monomorphic and a “-“ means no data.
